# The Application of the Extended Poincaré Plot in the Analysis of Physiological Variabilities

**DOI:** 10.3389/fphys.2019.00116

**Published:** 2019-02-19

**Authors:** Reem Satti, Noor-Ul-Hoda Abid, Matteo Bottaro, Michele De Rui, Maria Garrido, Mohammad R. Raoufy, Sara Montagnese, Ali R. Mani

**Affiliations:** ^1^UCL Division of Medicine, University College London, London, United Kingdom; ^2^Department of Medicine, University of Padova, Padova, Italy; ^3^Department of Physiology, Tarbiat Modares University, Tehran, Iran

**Keywords:** asthma, autocorrelation, cirrhosis, heart rate variability, Poincaré plot, survival, temperature variability

## Abstract

The Poincaré plot is a geometrical technique used to visualize and quantify the correlation between two consecutive data points in a time-series. Since the dynamics of fluctuations in physiological rhythms exhibit long-term correlation and memory, this study aimed to extend the Poincaré plot by calculating the correlation between sequential data points in a time-series, rather than between two consecutive points. By incorporating this so-called lag, we hope to integrate a temporal aspect into quantifying the correlation, to depict whether a physiological system holds prolonged association between events separated by time. In doing so, it attempts to instantaneously characterize the intrinsic behavior of a complex system. We tested this hypothesis on three different physiological time-series: heart rate variability in patients with liver cirrhosis, respiratory rhythm in asthma and body temperature fluctuation in patients with cirrhosis, to evaluate the potential application of the extended Poincaré method in clinical practice. When studying the cardiac inter-beat intervals, the extended Poincaré plot revealed a stronger autocorrelation for patients with decompensated liver cirrhosis compared to less severe cases using Pearson’s correlation coefficient. In addition, long-term variability (known as SD2 in the extended Poincaré plot) appeared as an independent prognostic variable. This holds significance by acting as a non-invasive tool to evaluate patients with chronic liver disease and potentially facilitate transplant selection as an adjuvant to traditional criteria. For asthmatics, employing the extended Poincaré plot allowed for a non-invasive tool to differentially diagnose various classifications of respiratory disease. In the respiratory inter-breath interval analysis, the receiver operating characteristic (ROC) curve provided evidence that the extension of the Poincaré plot holds a greater advantage in the classification of asthmatic patients, over the traditional Poincaré plot. Lastly, the analysis of body temperature from patients using the extended Poincaré plot helped identify inpatients from outpatients with cirrhosis. Through these analyses, the extended Poincaré plot provided unique and additional information which could potentially make a difference in clinical practice. Conclusively, the potential use of our work lies in its possible application of predicting mortality for the organ allocation procedure in patients with cirrhosis and non-invasively distinguish between atopic and non-atopic asthma.

## Introduction

The Poincaré plot is a scatter graph that visualizes the correlation between two consecutive data points in a time-series (i.e., *x*-axis: A_n_ versus *y*-axis: A_n+1_). In the past, it has extensively been used in the analysis of physiological fluctuations (e.g., heart rate variability analysis) and has enabled researchers to measure short- and long-term variability separately (Mani et al., 2009). Moreover, the Poincaré plot has the ability to assess the strength of correlation between consecutive points in a time-series (i.e., by calculating the Pearson’s correlation coefficient, r).

**FIGURE 1:**
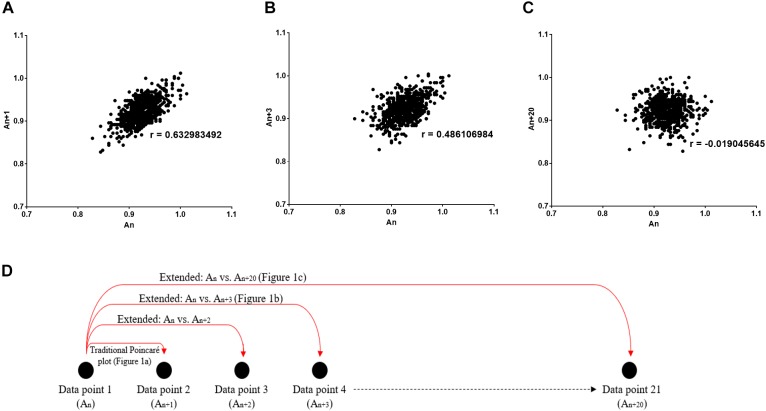
Poincaré plot shown which was used to study the correlation between R-R_n_ and R-R_n__+__k_ intervals. **(A)** *k* = 1 (conventional Poincaré plot), **(B)** *k* = 3, **(C)** *k* = 20 (extended Poincaré plot). *r* Describes the Pearson’s correlation coefficient. **(D)** Schematic diagram representing how the data points per extended Poincaré plot is derived.

In this study, we utilize the Poincaré plot by extending it, which essentially compares sequential data points in a time-series (i.e., *x*-axis: A_n_ versus *y*-axis: A_n__+__k_, where *k* can take any discrete value), rather than between two consecutive data points in the traditional plot (Figure 1A). Thus, due to the incorporation of this temporal aspect to the non-linear analysis, we can obtain the autocorrelation between two data points with a time lag. This can be considered as vital to clinical and physiological understanding, since a greater correlation between the present and past suggests the presence of memory in a time-series (Shirazi et al., 2013). In fact, in accordance to control theory, it is more difficult to manage a system which has a strong correlation with its past.

As physiological processes with stronger memory for a longer period of time are often deemed as less controllable (Shirazi et al., 2013; Mazloom et al., 2014; Ghafari et al., 2017), we expect the extended Poincaré plot to potentially act as an indicator of long-term correlation in a physiological time-series. Thereby, to test the credibility of our method, we used simulated noises to serve as our standard controls. An example of the latter, is the well-known memoryless phenomenon, white noise, where current events ‘forget their past’ in a random time-series. Contrariwise, if the current status of a system is more correlated, it relies on past events, though dependency could possibly be restricted to a limited number of past steps. In addition, we examined three physiological time-series, with the intention to provide a more advanced and comprehensive view on physiology. The clinical groups are as follows:

(1) Heart Rate Variability (HRV) data from patients with liver cirrhosis: Decreased HRV holds prognostic significance in chronic liver disease (Bhogal et al., 2018; Mani et al., 2009). A variety of different methods have been developed for complexity analysis of HRV including multiscale entropy and multifractal analysis (Peng et al., 1995; Costa et al., 2005; Zheng et al., 2010; Bian et al., 2012; Jiang et al., 2013; Song et al., 2013), however, recent studies revealed that the Poincaré plot provides better prognostic value in patients with liver disease in comparison with entropy and fractal indices of HRV (Bhogal et al., 2018). In this study, HRV is analyzed using the extended Poincaré plot to determine if the greater correlation between sequential inter-beat intervals predict mortality for non-invasive patient monitoring.
(2) Inter-breath interval (IBI) data from asthmatics: Asthma being a heterogeneous disease in nature, means there is difficulty in distinguishing between atopic and non-atopic as well as controlled and uncontrolled asthma non-invasively. It is of interest in this study to determine whether a strong autocorrelation in IBI time-series is identified in the most pathological form of asthma and of which specific stage of the lag in the extended Poincaré plot is best for diagnosing between the different forms that asthma can present as, as non-invasive tool.
(3) Body temperature fluctuations from patients with liver cirrhosis: Temperature is regulated by both circadian and homeostatic processes that intercalate during health and disease. There is evidence to suggest that the pattern of body temperature fluctuation is different in patients with cirrhosis in comparison with healthy individuals (Garrido et al., 2017). Extended Poincaré plot allows further insight into the dynamics of thermoregulation by unraveling the presence of autocorrelation in body temperature time-series.

## Materials and Methods

### Extended Poincaré Plot

As discussed before, the Poincaré plot is a scatter graph constructed from consecutive data points in a given time-series [*x*-axis: A_n_, *y*-axis: A_n__+__1_, (Figure 1A)]. In doing so, it provides information on correlation (by calculating the Pearson’s correlation coefficient, *r*) and an estimation of short- and long-term variability of a time-series. The latter parameters are defined by the standard deviation perpendicular to the line of identity (SD1) and the standard deviation parallel the line of identity (SD2) (Hsu et al., 2012).

Poincaré plots can be extended by considering a lag (defined by a number of steps, *k*) when calculating the correlation of a time-series. The extension of the Poincaré plot gives rise to the extended Poincaré plot whereby A_n_ is plotted against A_n__+__k_, where *k* steps can take any discrete integer (Figure 1B); and in this study the range *k* = {1, 2, 3, …, 20} was used (Figure 1C). By comparing the first A_n_ interval with subsequent A_n__+__k_ intervals (Figure 1D), the extended Poincaré plot quantifies internal serial correlation (i.e., autocorrelation) within a sequential physiological time-series (Brennan et al., 2001).

In this present study, the extended Poincaré plot analysis was carried out on 3 different physiological time-series, namely:

(a) Cardiac inter-beat R-R intervals (R-R) recorded from patients with chronic liver disease
(b) Respiratory IBI recorded from patients with asthma
(c) Body surface temperature (T) recorded from patients with chronic liver disease. Furthermore, three types of stimulated noise (white, pink and Brownian) were also assessed using the extended Poincaré plot. All physiological recordings were approved by regional ethics committee and conducted in accordance to the Declaration of Helsinki (Hong Kong Amendment) and Good Clinical Practice guidelines.

### Outcome Measures of the Extended Poincaré Plot

MATLAB programming language was used for the implementation of all algorithms used in this study (see Supplementary Material 1 for the scripts). Via these algorithms, for each lag (step, *k*) in the extended Poincaré plot, the Pearson correlation coefficient (*r*), SD1 and SD2 were calculated to produce a series of numbers corresponding to the outcomes in association with the extent of the lag (steps, *k*).

### Time-Series

#### Stimulated Noise

All stimulated noise, in the form of white, Brownian and pink, were produced via MATLAB (MathWorks, R2017b). Firstly, gaussian white noise was produced by a MATLAB embedded random generator to be used as negative control for comparing a random memoryless process with human data. Brownian noise, a stochastic process with one step memory, was created by the integration of white noise, to act as a positive control for the presence of autocorrelation in a time-series. Meanwhile, pink noise, a time-series with fractal like 1/f dynamics, was generated by a MATLAB function created by Hristo Zhivomirov (2017).

#### Study Cohort A: Heart Rate Variability Analysis in Patients With Cirrhosis

##### Patient population and ethics

All data recordings were in accordance to the recommendations and approval from the University Hospital of Padova Ethics Committees. Whilst all patients provided a written informed consent, 98 patients with liver cirrhosis [mean (±1 SD) age: 57 (±10.9) years] from the Department of Medicine outpatient clinic of the University of Padova from 29 June 2009 until 2 May 2011 were enrolled. Patient selection was based on their etiology, established through clinical, laboratory, radiological and histological findings. Whereas, severity was characterized by Pugh’s modification of the Child’s grading system (Child-Pugh) and the Model for End-Stage Liver Disease (MELD) scores (Peng et al., 2016). Eligibility was based on a previously described criterion (Shirazi et al., 2013). 74 patients were eligible for an 18-month follow-up (median: 12 months) [mean (±1SD) age: 56 (±10.8) years] to retrieve information on the occurrence of death or liver transplantation; whereby urgent transplants were given to those whom would be considered ‘dead’ on the day of transplantation and this data was used for survival analysis. The age-matched controls comprised of 35 healthy volunteers [mean (±1SD) age: 55 (±11.8) years].

##### Data collection

Initially, a 10-min single channel ECG was obtained using conventional ECG electrodes. The data was digitized at a sampling rate of 256 Hz and detected R peaks attained a R-R interval time-series, via an ad hoc computer program (Chart 5, AD-Instrument, Australia). By visual inspection, 8-min artifact-free continuous R-R interval sections were used for analysis.

##### Outcome measures

From the extended Poincaré plot, the parameters obtained were Pearson’s *r*, SD1 and SD2. In addition, Cox’s proportional hazards ratio was calculated in the survival analysis to statistically analyze if the latter indices predict mortality.

#### Study Cohort B: Respiration Inter-breath Intervals From Patients With Asthma

##### Patient population and ethics

The study for this cohort was approved by an institutional review board and the ethics committee at Tarbiat Modares University and all participants signed written informed consent prior to data collection. 40 age-matched men, aged between 21 and 39 years, participated in this study during June 2010 to February 2011, in accordance to the eligibility criteria defined by Raoufy et al. (2016), including 10 healthy volunteers and 30 asthmatics from the outpatient clinic of Masih Daneshvari Lung Hospital (Tehran, Iran). Here, asthma was categorized by whether or not it was controlled based on the National Asthma Education and Prevention Program (NAEPP) guidelines, thereby leading to further classifications of 10 patients as having controlled atopic asthma (CAA), 10 patients with uncontrolled atopic asthma (UAA), and 10 patients with uncontrolled non-atopic asthma (UNAA).

##### Data collection

Respiratory IBIs were recorded to create a time series as subjects laid supine for about 70 min, whilst continuous respiratory signals were collected using respiratory inductive plethysmography, as described by Raoufy et al. (2016).

##### Outcomes measures

Again, the Pearson’s *r*, SD1 and SD2 were attained from the extended Poincaré plot algorithm output. Furthermore, a receiver operating characteristic (ROC) curve was used to determine the sensitivity and specificity of whether the plots extension, demonstrated by the lag (steps, *k*), were appropriate or not for distinguishing between different asthma classifications.

#### Study Cohort C: Body Surface Temperature Fluctuations in Liver Cirrhosis

##### Patient population and ethics

This particular study was approved by Padova University Hospital ethics committee. All participants provided written informed consent. Fifty three patients were recruited, however, 45 subjects were used for analysis based on the exclusion criteria stated by Garrido et al. (2017). The classification of liver cirrhosis was identified using various pathological findings as described above in Section “Stimulated Noise.” Therefore, 12 were outpatients with cirrhosis, 12 were inpatients with cirrhosis, 11 were inpatients without cirrhosis and 10 were healthy volunteers.

##### Data collection

The temperature recordings were as described by Garrido et al. (2017). In brief, the proximal skin temperature was recorded using the iButton (model no. DS1922L-F5, Maxim Integrated, San Jose, CA, United States) with a sampling rate of one sample per 3 min, with a resolution of 0.0625°C. The temperature recordings by the loggers were carried out over 24 h.

##### Outcomes measures

The Pearson’s *r*, SD1 and SD2 for this given time-series were calculated.

### Statistical Analysis

Data was statistically analyzed by a two-way ANOVA with Tukey’s *post hoc* test. *P*-values less than 0.05 were considered statistically significant. Cox’s proportional hazards model was used to assess the prognostic value of the Poincaré plot indices in patients with liver disease. The ROC curve was used to study the diagnostic ability of each step (*k*) in predication of different classes of asthma (e.g., atopic from non-atopic and controlled from uncontrolled asthma). The area under the ROC curve is a measure of how well a parameter can distinguish between two diagnostic groups (e.g., atopic versus non-atopic asthma). When there is a perfect separation of the values of the two groups, i.e., there no overlapping of the distributions, the area under the ROC curve equals 1. When the parameter cannot distinguish between the two groups the area will be equal to 0.5. Area under the curve and its *p*-value (null hypothesis: area = 0.5) were calculated using IBM SPSS Statistics 24. The ample size for each cohort was calculated based on a 0.05 significance level (error type I) and 0.80 power (1-error type II).

## Results

Through the extension of the Poincaré plot, the lag affects the elliptical shape of the plot. As the lag increases, the linear correlation generally decreases whilst SD1 and SD2 change in an inversely proportional manner (Figure 1).

### Stimulated Noise

**FIGURE 2:**
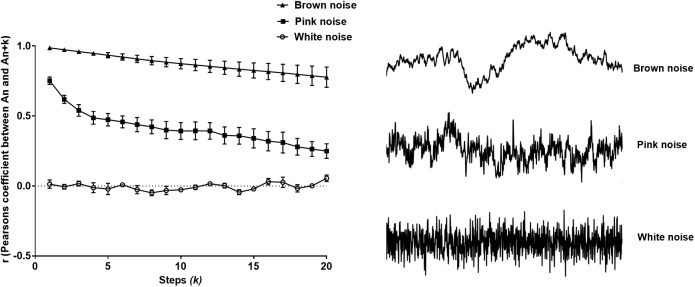
Pearson’s *r* from the extended Poincaré plot demonstrates the relationship between A_n_ and A_n__+__k_ (*k*_max_ = 20) in white noise (a random and memoryless process), pink noise (a process with 1/f dynamics) and Brown noise (stochastic process with memory).

Pearson’s *r* correctly identified models of colored noise, proving its reliability (Figure 2). With white noise being a completely memoryless phenomenon, r fluctuated around 0, since subsequent steps are not correlated with the first interval. Contrariwise, Brownian noise exhibited serial correlation, verifying that it holds memory. As we expected, pink noise lies in between the two latter extremes, namely white and Brownian noise (Halley and Kunin, 1999) as shown in Figure 2.

### Study Cohort A: Heart Rate Variability Analysis in Patients With Cirrhosis

#### Extended Poincaré Plot Outcome Measures

**FIGURE 3:**
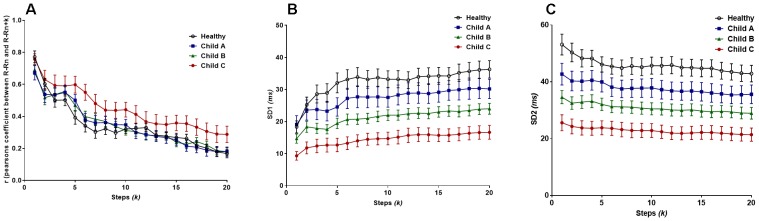
**(A)** Pearson’s *r* from the extended Poincaré plot demonstrates the relationship between R-R_n_ and R-R_n__+__k_ (*k*_max_ = 20) in healthy volunteers and patients with liver cirrhosis from the least severe (Child A), moderate (Child B) and in the most severe cases (Child C). **(B)** The SD1 calculated in the extended Poincaré plot is shown. **(C)** The SD2 calculated in the extended Poincaré plot is shown.

Regarding Pearson’s *r*, the two-way ANOVA confirmed a significant difference between at least one sub-population group (*F*_group_ = 28.63, *P* < 0.0001). It is clear that the most severe class of cirrhosis (Child C) is distinctly disparate from all other subjects in the study, by shifting toward the Brownian noise spectrum, illustrating a more correlated rhythm across lag-*r*, also known as the extended Poincaré plot (Figure 3A). In other words, Person’s *r* can only distinguish Child C patients from other groups. Even though there is a significant effect for *k* (F_lag_ = 53.5*, P* < 0.0001), there was no interaction between patient groups and lag across the range of values for *k* and thus, Pearson’s *r* exhibits the same trend across all groups in respect to *k*.

Figure 3B unveiled significant differences in the sample groups (*F*_group_ = 186.3, *P* < 0.0001) and subsequent steps, *k*, (*F*_lag_ = 5.462, *P* < 0.0001) for SD1. SD1 could separate different classes of cirrhosis with the lowest value being for Child C and the highest for healthy individuals as shown Figure 3B. SD1 increased in parallel with an increasing value of *k*, with the greatest gross increase in healthy individuals. However, there is no interaction between lag-*k* and the subpopulation groups since the variables are parallel to one another (two-way ANOVA).

Likewise, SD2 can also separate different classes of cirrhosis from the healthy group of individuals (Figure 3C). On the contrary, SD2 decreased by an average of 17.4% across all subjects as *k* approaches 20 (Figure 3B). There is a significant difference between groups (*F*_group_ = 234.9*, P* < 0.0001), however, there is no significant difference between the individual steps (*F*_lag_ = 1.536*, P* = 0.0641) (two-way ANOVA). Moreover, in respect to *r*, there is no significant difference in trend between the sample groups as *k* increases.

#### Survival Analysis

Study participants were followed up for 18-months post-ECG recording to obtain their survival outcomes for use in our mortality studies. Cox’s regression analysis was used to assess whether HRV indices predicated mortality. The hazard ration [Exp(β)] was calculated. In this analysis, Exp(β) = 1, which indicates no effect in predicating mortality, whilst Exp(β) < 1 or Exp(β) > 1 indicate whether the index is either protective or hazardous respectively, when predicating survival.

**FIGURE 4:**
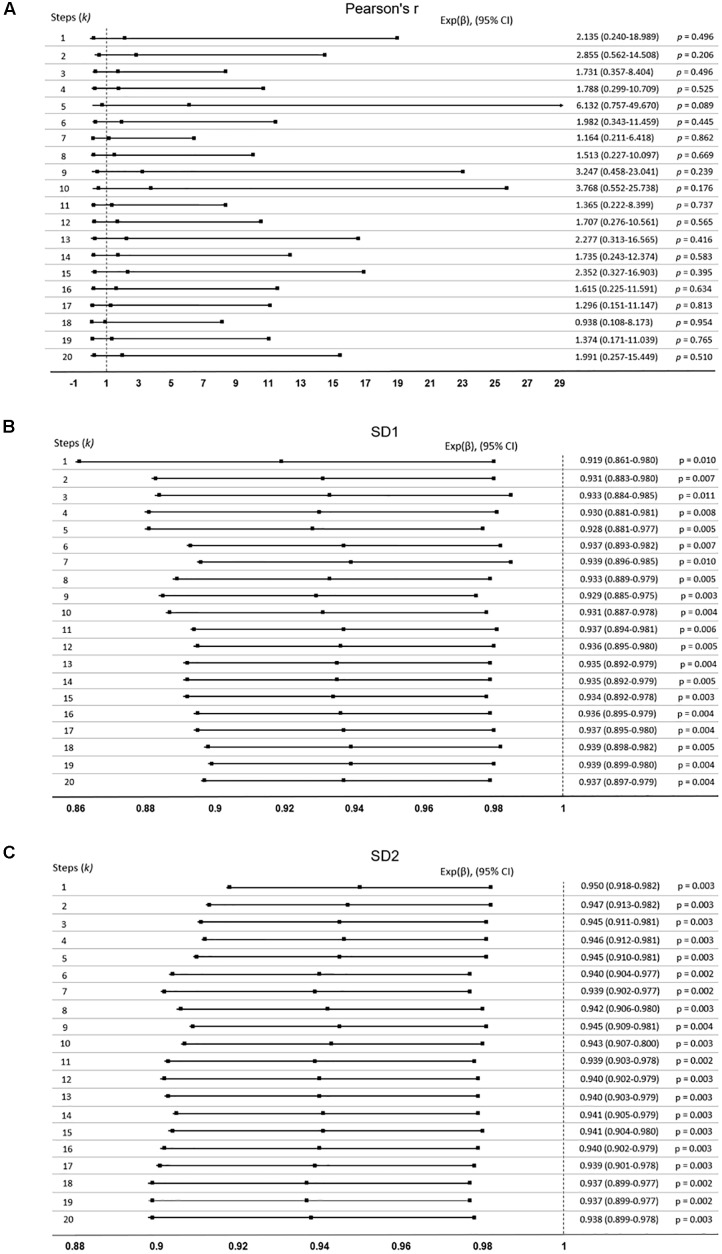
Cox-proportional hazards model was used to determine if the extended Poincaré plot parameters predict mortality in patients with cirrhosis. Data expressed as: Exp(β), (95% confidence interval, CI). If indices predict mortality, the confidence interval will not cross Exp(β) = 1. **(A)** Pearson’s *r* did not predict mortality, whilst **(B)** SD1, and **(C)** SD2 did.

Pearson’s *r* presented no prognostic capacity, as it did not predict the 18-month survival outcome (*P* > 0.05) and all the 95% confidence intervals crossed the no effect size. The lowest *p*-value was 0.089 (95% confidence interval: 0.757–49.670) at *k* = 5 (Figure 4A).

Nevertheless, Figure 4B,C shows SD1 and SD2 respectively, both of which significantly predict mortality with an increasing *k* (*P* < 0.05). SD2 generally was more significant than SD1 throughout the lag-*k*. As expected the indices of the severity of liver dysfunction (MELD and Pugh) could robustly predict mortality (*P* < 0.0001). In order to see whether or not the prognostic value of SD1 and SD2 depend on the severity of liver dysfunction, we used a multivariate Cox’s regression analysis. It appears that the ability of SD1 to predict mortality is dependent on disease severity, across all steps, since *P* < 0.0001 for the MELD score, but the *p*-value was insignificant for the sole use of SD1 without consideration of disease severity (*P >* 0.05, Table 1A). However, SD2 has a greater ability to predict mortality and also independently to the MELD score, though varyingly and inconsistently across lag-*k* (Table 1B).

**Table 1:** Bivariate Cox-regression to determine SD1 and SD2’s dependency on the MELD score when predicting mortality (*k*^∗^ = independent).

Steps (*k*)	Exp(β)	95% CI	*P*-value	Ex p(β)	95% CI	*P*-value
**(A)**	**MELD**			**SD1**		
1	1.154	(1.070-1.244)	0.000	0.956	(0.897–1.018)	0.162
2	1.152	(1.069–1.241)	0.000	0.959	(0.912–1.008)	0.103
3	1.156	(1.072–1.246)	0.000	0.963	(0.917–1.012)	0.138
4	1.153	(1.070–1.243)	0.000	0.959	(0.911–1.009)	0.105
5	1.148	(1.066–1.236)	0.000	0.954	(0.907–1.002)	0.063
6	1.152	(1.068–1.242)	0.000	0.964	(0.921–1.010)	0.122
7	1.154	(1.070–1.244)	0.000	0.966	(0.923–1.011)	0.138
8	1.151	(1.067–1.241)	0.000	0.960	(0.916–1.006)	0.089
9	1.148	(1.065–1.237)	0.000	0.955	(0.911–1.002)	0.058
10	1.148	(1.066–1.237)	0.000	0.956	(0.913–1.002)	0.059
11	1.151	(1.069–1.241)	0.000	0.962	(0.920–1.006)	0.089
12	1.151	(1.068–1.240)	0.000	0.961	(0.919–1.004)	0.076
13	1.150	(1.067–1.239)	0.000	0.959	(0.918–1.003)	0.068
14	1.150	(1.068–1.239)	0.000	0.960	(0.918–1.004)	0.071
15	1.149	(1.067–1.238)	0.000	0.959	(0.918–1.002)	0.062
16	1.150	(1.067–1.240)	0.000	0.961	(0.921–1.004)	0.073
17	1.152	(1.069–1.241)	0.000	0.962	(0.921–1.004)	0.079
18	1.153	(1.070–1.242)	0.000	0.963	(0.923–1.005)	0.083
19	1.151	(1.068–1.240)	0.000	0.963	(0.923–1.004)	0.073
20	1.150	(1.068–1.239)	0.000	0.962	(0.922–1.003)	0.069

**(B)**	**MELD**			**SD2**		
1^∗^	1.148	(1.066–1.236)	0.000	0.967	(0.935–0.999)	0.043
2^∗^	1.148	(1.066–1.236)	0.000	0.965	(0.931–0.999)	0.046
3^∗^	1.147	(1.065–1.235)	0.000	0.964	(0.930–0.999)	0.046
4	1.148	(1.065–1.237)	0.000	0.966	(0.932–1.001)	0.057
5	1.148	(1.066–1.237)	0.000	0.965	(0.931–1.001)	0.057
6^∗^	1.146	(1.065–1.234)	0.000	0.959	(0.924–0.997)	0.032
7^∗^	1.145	(1.064–1.233)	0.000	0.959	(0.922–0.996)	0.031
8^∗^	1.147	(1.065–1.235)	0.000	0.962	(0.927–0.999)	0.044
9	1.149	(1.067–1.237)	0.000	0.964	(0.929–1.001)	0.057
10	1.149	(1.066–1.237)	0.000	0.963	(0.927–1.001)	0.056
11^∗^	1.147	(1.065–1.235)	0.000	0.960	(0.923–0.998)	0.041
12^∗^	1.147	(1.065–1.235)	0.000	0.961	(0.924–1.000)	0.048
13	1.147	(1.065–1.236)	0.000	0.962	(0.925–1.001)	0.053
14^∗^	1.148	(1.066–1.236)	0.000	0.962	(0.925–1.000)	0.050
15	1.148	(1.066–1.236)	0.000	0.962	(0.925–1.001)	0.053
16^∗^	1.147	(1.066–1.235)	0.000	0.960	(0.923–0.999)	0.047
17^∗^	1.146	(1.064–1.234)	0.000	0.960	(0.922–0.999)	0.044
18^∗^	1.145	(1.064–1.233)	0.000	0.958	(0.920–0.998)	0.041
19^∗^	1.146	(1.064–1.234)	0.000	0.959	(0.920–0.999)	0.046
20	1.147	(1.065–1.235)	0.000	0.960	(0.921–1.000)	0.051

***(A)** SD1 depends on the MELD score to predict mortality but **(B)** SD2 does not.*

### Study Cohort B: Respiration Inter-Breath Intervals From Patients With Asthma

#### Extended Poincaré Plot Outcome Measures

**FIGURE 5:**
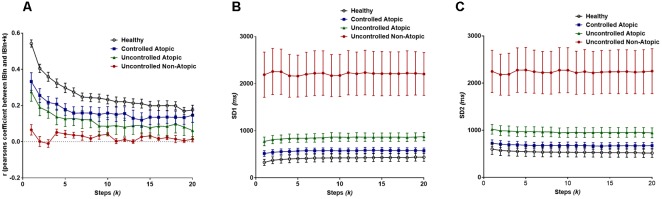
**(A)** Pearson’s *r* from the extended Poincaré plot demonstrates the relationship between IBI_n_ and IBI_n__+__k_ (*k*_max_ = 20) in healthy volunteers and patients with different types of asthma. **(B)** The SD1 calculated in extended Poincaré plot is shown. **(C)** The SD2 calculated in extended Poincaré plot is shown.

Pearson’s *r* demonstrated that there is significant difference between the lags (steps, *k*) (*F*_lag_ = 8.54, *P* < 0.0001), as well as the different classification groups of asthma (*F*_group_ = 170, *P* < 0.0001). As seen in Figure 5A, the healthy patients have the greatest change in autocorrelation and the least change is seen in the most severe form of asthma, uncontrolled non-atopic asthma. Yet, there is no interaction between the lag and the subgroups of asthma.

For both SD1 and SD2, there is a clear distinction between at least one patient group, i.e., again uncontrolled non-atopic asthma, from the rest of the study population (*F*_group_ > 200*, P* < 0.0001). However, there is no significant difference between each step, *k*, and the interaction also presents as significant (Figure 5B,C).

#### Diagnostic Value of the Extended Poincaré Plot in Different Classes of Asthma

Using the area under the ROC curve, Pearson’s r had the best sensitivity and specificity at distinguishing between atopic and non-atopic asthma, at *k* = 3 (*P* < 0.0001, The area under the ROC curves are shown in Supplementary Materials 2a,b). In addition, *k* = 2 and *k* = 12 had the most significant result in distinguishing between controlled and uncontrolled asthmatic patients (*P* = 0.002).

**FIGURE 6:**
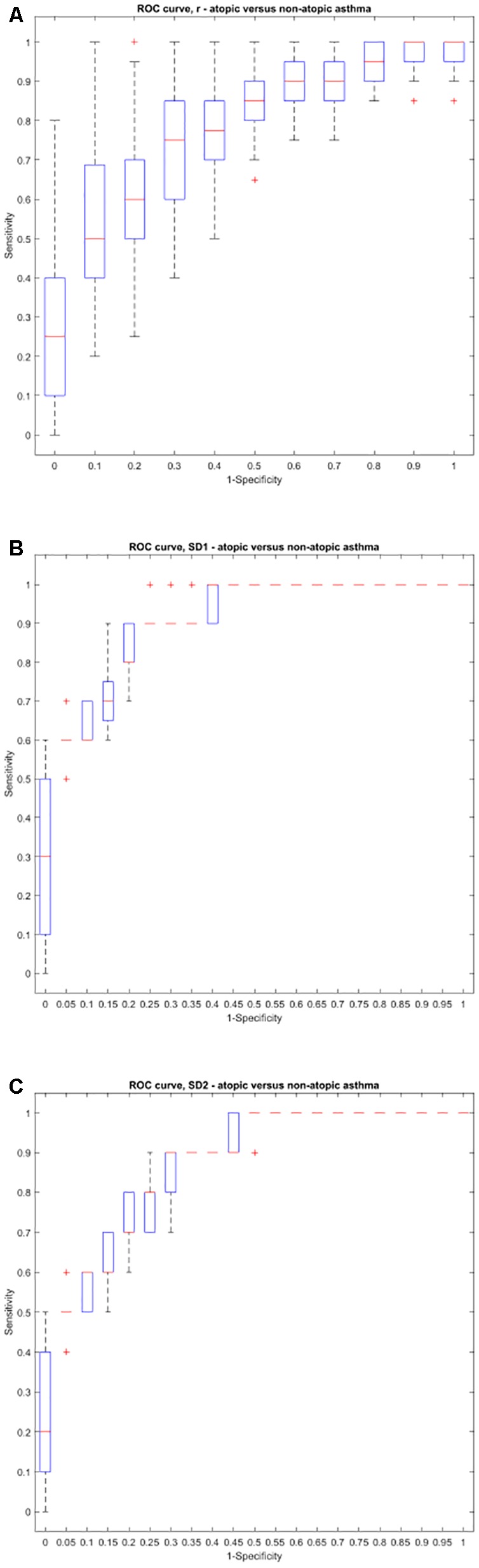
Boxplots representing the ROC curves that were used to study the diagnostic ability of *r*, SD1, or SD2 in different steps to diagnose atopic from non-atopic asthma. The area under the ROC curve is a measure of how well a parameter such as Pearson’s *r* **(A)**, SD1 **(B)**, or SD2 **(C)** can distinguish between atopic and non-atopic asthma. The area under the curves for each step (*k*) can be found in Supplementary Material 2.

**FIGURE 7:**
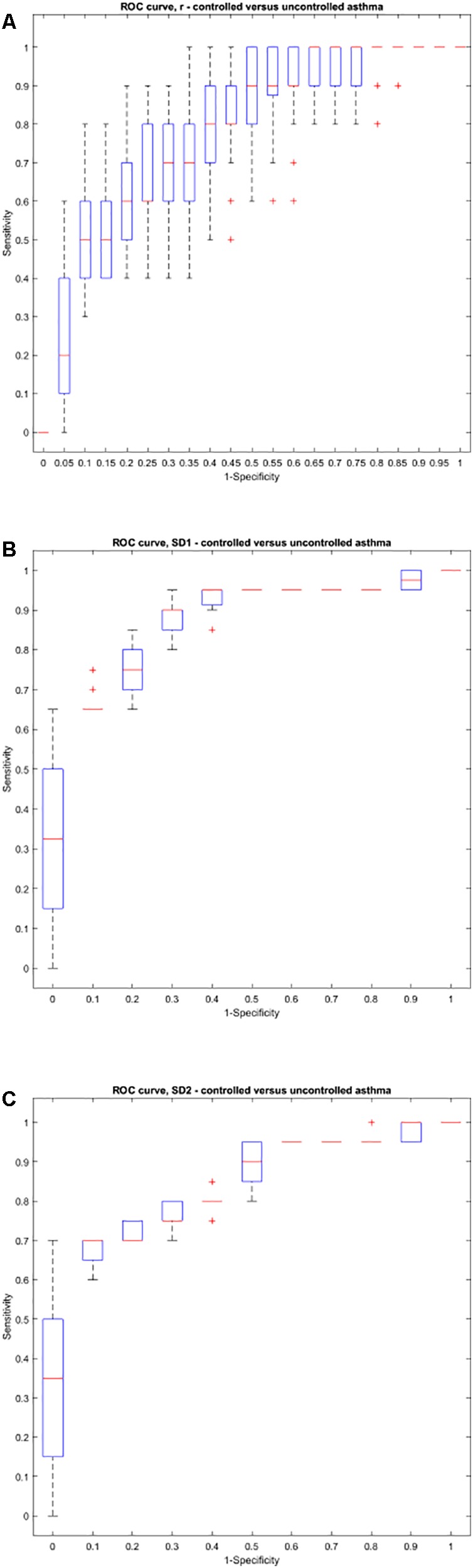
Boxplots representing the ROC curves that were used to study the diagnostic ability of *r*, SD1, or SD2 in different steps to diagnose controlled from uncontrolled asthma. The area under the ROC curve is a measure of how well a parameter such as Pearson’s *r* **(A)**, SD1 **(B)**, or SD2 **(C)** can distinguish between controlled and uncontrolled asthma. The area under the curves for each step (*k*) can be found in Supplementary Material 2.

Interestingly, both SD1 and SD2 had consistent diagnostic ability across all steps (*k*) for classifying atopic from non-atopic and uncontrolled from controlled asthma (Supplementary Materials 2a–c). Boxplots depicting the ROC curves for different *k* values are demonstrated in Figure 6, 7).

### Study Cohort C: Body Surface Temperature Fluctuations in Liver Cirrhosis

#### Extended Poincaré Plot Outcome Measures

**FIGURE 8:**
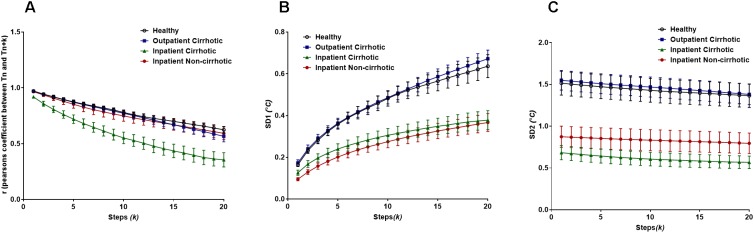
**(A)** Pearson’s *r* from the extended Poincaré plot demonstrates the relationship between body temperature fluctuations, T_n_ and T_n__+__k_ (*k*_max_ = 20) in healthy volunteers, inpatients and outpatients. **(B)** The SD1 calculated in the extended Poincaré plot is shown. **(C)** The SD2 calculated in the extended Poincaré plot is shown.

For the temperature fluctuation data from subsets of cirrhotic patients, Pearson’s *r* demonstrated to profoundly differentiate cirrhotic inpatients from other groups (cirrhotic outpatients, non-cirrhotic inpatients and lastly healthy individuals, Figure 8A). The two-way ANOVA analysis revealed there are significant differences between patient groups (*F*_group_ = 122.1, *P* < 0.0001) and steps (*k*) (*F*_lag_ = 40.55, *P* < 0.0001). Yet, there is no interaction between the two since all patient groups follow a declining trend across the lag.

As shown in Figure 8B, the SD1 analysis of the data enabled us to distinguish between inpatients and outpatients (including healthy volunteers). Similarly, to Pearson’s *r*, there are significant differences between patient groups (*F*_group_ = 221.9, *P* < 0.0001) and steps (*k*) (Flag = 41.81, *P* < 0.0001), however, there is no interaction between the two (F_interaction_ = 1.281, *P* = 0.0840).

Nevertheless, the lag-SD2 (which is depicting long-term variability) illustrates a slight declining trend that also separates inpatients from outpatients. Though SD2 uniquely allows for the segregation of cirrhotic inpatients and non-cirrhotic inpatients, across the increasing lag (Figure 8C). In fact, there is a significant difference between patient groups (*F*_group_ = 272.6, *P* < 0.0001) but not between steps (*k*), meaning that the declination trend is not significant. With that being said, there is no interaction between patient groups and steps (*k*).

## Discussion

In this present study, the integrity of homeostasis within three different physiological time-series were analyzed by the extended Poincaré plot, by measuring their internal serial correlation. Through the use of Pearson’s *r*, the extent of correlation in a given physiological time-series can be quantified, whereby a greater value of correlation conveyed prolonged memory and subsequently impaired homeostasis, as it conveys less adaptable state (Mazloom et al., 2014). With that being said, the implementation of Pearson’s *r*, from the extended Poincaré plot has not previously been used to quantify memory in a physiological time-series in the same context and with the same intention. In addition, to illustrate reliable controls for this study, stimulated noises were used for referencing the scale of changes in the time-series.

Firstly, HRV was analyzed in patients with liver cirrhosis. The magnitude of the inter-beat interval fluctuations in HRV is a hallmark of disease, where lessened HRV acts as a negative predictor of patient outcome in liver cirrhosis (Mani et al., 2009; Bhogal et al., 2018). In consensus with Shirazi et al.’s method, Pearson’s r revealed that severely cirrhotic patients exhibited prolonged memory (Shirazi et al., 2013). Furthermore, both extended-SD1 and SD2 were able to distinguish between different severities of liver cirrhosis and from their healthy controls. Though, unlike Pearson’s *r*, SD1 and SD2 showed prognostic capacity. The inability of Pearson’s *r* to predict mortality was not due to suboptimal study design, as the minimum sample size was exceeded. Yet, this result verified that enhanced cardiac autocorrelation was not the main cause of death in liver cirrhosis, which is supported by the fact that patients are more likely die from other complications, such as variceal bleeding or infection/sepsis (O’Brien et al., 2012) and not severe congestive heart failure (Lee and Liu, 2007). Pearson’s r in the extended Poincaré plot did not hold prognostic capacity. However, it could possibly be used in the future to non-invasively determine liver cirrhosis complications, like cirrhotic cardiomyopathy, which is more prevalent in decompensated liver cirrhosis. It is also noteworthy to mention, that till-date complications like cirrhotic cardiomyopathy have no well-established guideline for diagnosis, even though such complications negatively impact the prognosis of liver cirrhotic patients undergoing stressful events, such as liver transplantation or transjugular intrahepatic portosystemic shunts (TIPS) (Lee and Liu, 2007; Møller and Lee, 2018).

We also calculated SD2 and SD1 in the extended Poincaré plot by computing the standard deviation of variations along or perpendicular to the line of identity. SD1 in the traditional Poincaré plot is linked with short-term variability and respiratory sinus arrhythmia (Haddadian et al., 2013). Introducing a lag in the Poincaré plot shifts short-term variability toward longer variations. Our results revealed that cirrhotic patients had a significant reduction in both SD1 and extended-SD1 in comparison with healthy controls. Extended-SD1 could also predict survival in this patients’ population. However, the ability of extended-SD1 to predict mortality was dependent on the MELD score and thus does not add more information to the MELD score in prediction of survival. SD2 measures long-term fluctuations in a time-series and within the context of HRV is linked with thermoregulation and baroreflex loop. SD2 surprisingly predicted mortality independent to the MELD score in liver cirrhosis. The MELD score is currently the main clinical criteria for the assessment of liver dysfunction for organ allocation (i.e., in liver transplantation). The extended Poincaré plot provides indices that predict mortality independent of the MELD score. Thus, the Poincaré plot has the potential to be used concomitantly with the MELD score, to increase the accuracy of finding the most suitable organ recipient for organ allocation to patients awaiting liver transplantation. This would not be the first adjuvant, since the MELD score has been jointly used with physio-markers (e.g., EEG) which have been shown to assist mortality prediction in patients with cirrhosis (Montagnese et al., 2015). The advantage of the Poincaré plot is that it can be easily calculated by incorporating a simple computational script to bedside monitors. Furthermore, this incorporation may allow for assisting patient selection for stressful procedures such as liver transplantations or TIPS, as it identifies which patient is most likely to survive the procedure, Thereby, the extended Poincaré plot can potentially add value in assisting patient transplant selection, by combatting the subjective assessment of clinicians. However, the reason behind the prognostic capacity of long-term HRV is unknown, though it is thought to be linked with thermoregulation and, in fact, recent reports indicate that thermoregulation is impaired in cirrhosis (Garrido et al., 2017; Mani et al., 2018). Nevertheless, the association between SD2 and core body temperature fluctuations in cirrhosis remains unstudied and requires further investigation.

Secondly, IBI data was analyzed from patients with different classes of asthma to explore the diagnostic capability of the extended Poincaré plot. The extended Poincaré plot indices, Pearson’s *r*, SD1 and SD2, all appeared to distinguish between controlled and uncontrolled, as well as between atopic and non-atopic asthma. An important goal of research in the disease, asthma, is to identify subgroups that respond well to different types of treatments. Currently, there is no simple test available to identify patients who do not respond to common therapies (uncontrolled asthma). Recent investigations in this field are focused on analyzing bronchoalveolar lavage and its specific cellular/cytokine profile or plasma/urinary biomarkers (Wedes et al., 2011; Hosoki et al., 2015). Our results indicate that the extended Poincaré plot’s analysis of patients’ IBI time-series data can separate different subgroups of patients with asthma without the need for clinical invasive means. Pearson’s *r* revealed uncontrolled non-atopic asthma to be close to the memoryless phenomenon, white noise, which seems to maintain the weakest correlation across the lag (weaker autocorrelation). The correlation coefficient *r*, appeared to also hold a non-invasive diagnostic value when it comes to differentiating between uncontrolled and -controlled asthma as well as between atopic and non-atopic asthma, especially at greater steps of lag (*k* > 1). The latter, is very important at supporting the fact that the extended Poincaré plot holds more temporal information, as well diagnostic significance in comparison to the traditional Poincaré plot. Furthermore, SD1 and SD2 showed similar results, by again easily detecting uncontrolled non-atopic asthma from the other subsets of asthma. The diagnostic ability of the extended Poincaré plot across the lag was consistent, and was best portrayed by quantifying correlation to assess the ‘memory’ of the system and its time-series.

The ROC curve was also implemented to optimize the sensitivity and specificity of the extended Poincaré plot in the diagnosis of different types of asthma. Pearson’s *r* had the best sensitivity and specificity at distinguishing between atopic and non-atopic asthma, especially at *k* = 3. Furthermore, *k* = 2 and *k* = 12 had the most significant result in distinguishing between controlled and uncontrolled asthma. These results are promising and also go along with our previous report on the classification of asthma based on respiratory fluctuation analysis (Raoufy et al., 2016). In addition, Frey et al. reported that fluctuation analysis of airway function provides a quantitative basis for objective risk prediction of asthmatic episodes and also for evaluating the effectiveness of therapy (Frey et al., 2005). We hypothesize that the incorporation of the extended Poincaré plot analysis with routine spirometry/plethysmography could be used in future to allocate the best therapy for patients and therefore to ultimately improve patient outcomes. Thus, this allows us to believe that the impact of the extended Poincaré plot in asthma lies in being able to provide information computationally, which currently is not fulfilled. However, a multicenter study with a larger cohort of patients is required to fully test this hypothesis.

Finally, the body surface temperature fluctuation data obtained from patients with liver cirrhosis revealed interesting results. Pearson’s r from the extended Poincaré plot easily distinguished inpatients with liver cirrhosis from all other subsets of cirrhotic patients, especially with the increasing lag. The extended Poincaré plot showed that there was a decrease in autocorrelation, suggesting that these temperature fluctuations seemed to be less correlated with previous temperature recordings in the same time series. This may tie in with systemic inflammation that occurs with liver cirrhosis, patients of which are usually hospitalized. Loss of autocorrelation in body temperature fluctuations goes along with increased entropy and thus disorder of this time-series; a phenomenon that has already been reported in patients and animal model of cirrhosis (Garrido et al., 2017; Mani et al., 2018). It appears that the extended Poincaré plot depicts that hospitalized patients with cirrhosis, tend toward the white noise side of the spectrum, which has high degree of entropy as it’s a memoryless phenomenon, therefore has no degree of regularity, the inverse of which is used to quantify entropy. In addition, we also observed a significant inverse correlation between Person’s r and the sample entropy of the temperature time-series (data not shown).The weaker autocorrelation and increased entropy of the body temperature fluctuations observed, may also indicate a stronger engagement of the thermoregulatory system due to systemic inflammation (Garrido et al., 2017). We hypothesize that body temperature fluctuation analysis could also be used for screening of cirrhotic patients who have developed inflammation-related complications (e.g., infection). It appears that the variability analysis gives more information about the thermoregulatory system, than its absolute value (Papaioannou et al., 2012). Detection of fever has historically been used for screening of infection, yet, none of the patients in this study had fever; however, temperature variability analysis can distinguish between inpatients and outpatients with chronic liver disease. Future studies will pave the way to test the potential application of temperature fluctuation analysis in the detection of systemic inflammatory response syndrome in patients with cirrhosis.

Both SD1 and SD2 in the extended Poincaré plot analysis of body temperature fluctuations are higher in volunteers who were not hospitalized (i.e., healthy controls and outpatients with cirrhosis). Since hospitals have a temperature-controlled environment and patients spend most of their time at their bedside bed, it is expected to see less short-term and long-term temperature variability in hospitalized patients, in comparison to outpatients and healthy controls. Interestingly both inpatient groups (cirrhotic and non-cirrhotic) exhibit similar SD1 and SD2 parameters, whilst their Pearson’s correlation coefficient is significantly different. This clearly shows that the loss of autocorrelation in cirrhotic inpatients is not related to less variation in their temperature profile.

The Poincaré plot has numerous applications in different disciplines and is known by other names such as, return maps, the Poincaré return plot or Lorenz plot in the mathematics and physics community. The classical application of return maps is in detecting non-linearity in deterministic one-dimensional systems (Kaplan et al., 1996). First-return Poincaré plots have been used extensively in medicine, not to detect non-linearity but to unravel the extent of short-term and long-term variability in the time-series by calculating the standard deviation along the axes of the plot (e.g., SD1 and SD2). Our aim in the study was to examine the application of these simple return maps as a first attempt to quantify autocorrelation which we then used to determine SD1 and SD2. We used the term “extended Poincaré plot” to show that we have extended the traditional Poincaré plot by looking at the autocorrelation with a lag as shown in Figure 1. As the extended Poincaré plot assesses autocorrelation in a time-series, a higher degree of autocorrelation suggests the presence of an autoregressive process. An in-depth analysis can provide us more information about the order of such a process (Ramsey, 1974). In a first order autoregressive process [AR(1)], the current value of the process is based on the immediately preceding value, and we expect to observe the following behavior:

$$A_{\text{n}}+1=\rho A_{\text{n}}+\xi [\text{Equation}1\;:\text{ AR}(1)\text{process}]$$

Where *ξ* is a random noise, and ρ is the parameter of the model and relates to the extent of the correlation (−1 < ρ < 1). In higher order autoregressive processes, we observe more complex behavior. For example, in a second order autoregressive process [AR(2)], the current value is based on the previous two values:

$$A_{\text{n}+2}=\rho_{1}A_{\text{n}+1}+\rho_{2}A_{\text{n}}+\xi '[\text{Equation}2\;:\text{ AR}(2)\text{process}]$$

Interestingly, in an AR(1) process, A_n__+__2_ might still be correlated with A_n,_ as the expansion of Equation 1 gives rise to Equation 3:

$$A_{\text{n}+2}=\rho^{2}A_{\text{n}}+\rho \xi +\xi '(\text{Equation}3)$$

This shows that in an AR(1) process, the extent of correlation between A_n__+__2_ and A_n_ depends on ρ^2^ (ρ squared) which is less than ρ itself. Therefore, observing a correlation between A_n__+__2_ and A_n_ does not rule out a first order autocorrelation. A systematic way to determine the order of an autoregressive process is using the partial autocorrelation function (Ramsey, 1974). We used this to analyze (see Supplementary Material 3) the three different types of physiological time-series and our results showed that in most of the time-series that we analyzed, we encounter an AR(1) process. The R-R interval data exhibit a low order autoregressive process, with some degree of oscillation, which might probably be related to the effect of respiration (respiratory sinus arrhythmia) on the R-R interval variation data as shown in Supplementary Material 3, the extended Poincaré plot can be further analyzed to show the partial autocorrelation of a physiological time-series in addition to extended SD1 and SD2. Our results show that the order of the autoregressive process was not markedly different between groups. Thus, the value of partial autocorrelation analysis in physiological time-series awaits further investigation.

The Poincaré plot provides a simple visual method that can be easily applied to bedside tests to aid diagnosis or treatment (e.g., in the organ allocation procedure in cirrhosis). In addition to this classic analytical method, other state of the art visual analytical methods do exist which are used for analysis of physiological time-series such as the recurrence plot (Webber and Zbilut, 1994; Marwan et al., 2002, 2007; Pham, 2018). The recurrence plot, it is a visual way of characterizing the recurrence, regularity and the order of the time series, from which recurrence quantification analysis can be used to determine the entropy (Marwan et al., 2007). In a recent report we showed that entropy analysis does not yield significant results in predicting mortality nor in clinical application in patients with liver disease (Bhogal et al., 2018). Therefore, we did not pursue the recurrence plot to use in our study on patients with liver disease. However, future studies are required to assess the application of this state of the art method along with other novel methods in different clinical settings.

## Conclusion

Our study showed the potential application of the extended Poincaré plot in analyzing various physiological time-series to unravel the resiliency and tolerance of a system that operates against environmental, physiological and pathological challenges. As some variability and plasticity in physiological rhythms are unavoidable, due to the balance between adaptation and memory, physiological controllability can be considered as a critical aspect of homeostasis, to enhance organismal function. It appears that the extension of the Poincaré plot can be applied to current health care challenges, such as the diagnosis of uncontrolled asthma, prediction of survival and assessment of thermoregulation in patients with liver disease. As robust as this present study appears, it lacked multicenter study heterogeneity, such as varying etiology. Even though it is alleged that predictors of mortality derived from HRV are irrespective of the etiology (Saraiva et al., 2001), it would be more appropriate to perform a multicenter study to momentously promote heterogeneity; as well as increasing the sample size, to further support the true clinical ability of Pearson’s *r*. Another limitation of this study is that, mathematical models and correlation-based approaches do not provide full enlightenment of a system’s behavior, as it oversimplifies critical physiological concepts, by statistically discriminating against stochastic processes. Moreover, Pearson’s *r* was chosen for the linearity seen in Poincaré plot and its extension as it assumes that the variables in the extended Poincaré plot exhibit a linear relationship, which limits validity if it does not yet, it does nonetheless establish immediate new knowledge, which can potentially be used to solve unmet clinical needs.

## Author Contributions

RS, MB, SM, and AM contributed in work conception. MDR, MR, MG, and MB curated the data. RS and N-U-HA performed the formal analysis. RS, N-U-HA contributed the software. SM and AM supervised the work. RS wrote the original draft. N-U-HA, SM, and AM wrote, review, and edited the manuscript.

## Conflict of Interest Statement

The authors declare that the research was conducted in the absence of any commercial or financial relationships that could be construed as a potential conflict of interest.
