# Corrigendum: The Application of the Extended Poincaré Plot in the Analysis of Physiological Variabilities

**DOI:** 10.3389/fphys.2019.00669

**Published:** 2019-05-28

**Authors:** Reem Satti, Noor-Ul-Hoda Abid, Matteo Bottaro, Michele De Rui, Maria Garrido, Mohammad R. Raoufy, Sara Montagnese, Ali R. Mani

**Affiliations:** ^1^UCL Division of Medicine, University College London, London, United Kingdom; ^2^Department of Medicine, University of Padova, Padova, Italy; ^3^Department of Physiology, Tarbiat Modares University, Tehran, Iran

**Keywords:** asthma, autocorrelation, cirrhosis, heart rate variability, Poincaré plot, survival, temperature variability

An author name was incorrectly spelled as “Mohammad R. Rauofy.” The correct spelling is **“**Mohammad R. Raoufy.”

The authors apologize for this error and state that this does not change the scientific conclusions of the article in any way. The original article has been updated.

