# Supplementary material for: The Application of the Extended Poincaré Plot in the Analysis of Physiological Variabilities
Source: Front Physiol. 2019 Feb 19;10:116. doi: 10.3389/fphys.2019.00116 (PMC6390508; doi:10.3389/fphys.2019.00116)

Supplementary materials

Supplementary material 1. MATLAB code for calculating the extended Poincaré plot indices i.e. Pearson's correlation coefficient (r), SD1 and SD2. Let A be a matrix which corresponds to a physiological time-series such as inter-beat interval, inter-breath interval or body temperature. This script can be save as a MATLAB function (extpoinc.m). Inputs: A (time-series) and k (maximum lag). Outputs: Pearson's r (r), P-value for the correlation coefficient (P), SD1 and SD2.

```
function [r,P, SD1, SD2] = extpoinc(A,k) % MATLAB function
for calculating the extended Poincaré plot indices.
Inputs: A (a time-series) and k (maximum steps/lags in the
extended Poincare' plot). Outputs: Pearson's r (r), P-
value for the correlation coefficient (P), SD1 and SD2.

n = size (A);
r = NaN(k,1);
P = NaN(k,1);
SD1 = NaN(k,1);
SD2 = NaN(k,1);

for i=1:k

     X = A(1:n-i);
     Y = A(i+1:n);
     [cc,p] = corrcoef(X,Y);
     r (i,1) = cc(1,2);
     P (i,1) = p(1,2);
     SD1 (i,1)= std((X - Y)./sqrt(2));
     SD2 (i,1)= std((X + Y)./sqrt(2));
end
```

Supplementary material 2. Receiver operating characteristic (ROC) curves used to evaluate the diagnostic capability of the extended Poincaré plot indices to differentiate between atopic and non-atopic asthma as well as controlled and uncontrolled asthma. (a) Pearson's correlation coefficient, r. (b) SD1 (c) SD2. (*optimal steps for disease classification)

(a)	Atopic vs non-atopic asthma			Controlled vs uncontrolled asthma		
Steps (k)	Area under the curve	Standard error	Asymptomatic Significance	Area under the curve	Standard error	Asymptomatic Significance
1	0.920	0.050	0.000	0.795	0.084	0.009
2	0.965	0.029	0.000	0.860	0.077	0.002*
3	0.980	0.023	0.000*	0.835	0.078	0.003
4	0.835	0.080	0.003	0.790	0.087	0.011
5	0.865	0.082	0.001	0.855	0.076	0.002
6	0.805	0.081	0.007	0.705	0.110	0.071
7	0.780	0.091	0.014	0.715	0.096	0.059
8	0.875	0.069	0.001	0.795	0.082	0.009
9	0.715	0.100	0.059	0.735	0.095	0.039
10	0.720	0.093	0.053	0.815	0.082	0.006
11	0.830	0.081	0.004	0.815	0.077	0.006
12	0.815	0.079	0.006	0.860	0.070	0.002*
13	0.825	0.075	0.004	0.720	0.105	0.053
14	0.660	0.106	0.159	0.630	0.111	0.253
15	0.665	0.105	0.147	0.745	0.097	0.031
16	0.810	0.079	0.006	0.800	0.082	0.008
17	0.745	0.090	0.031	0.790	0.083	0.011
18	0.795	0.092	0.009	0.755	0.089	0.025
19	0.810	0.080	0.006	0.785	0.085	0.012
20	0.725	0.093	0.048	0.825	0.082	0.004

(b)	Atopic vs non-atopic asthma			Controlled vs uncontrolled asthma		
Steps (k)	Area under the curve	Standard error	Asymptomatic Significance	Area under the curve	Standard error	Asymptomatic Significance
1	0.920	0.048	0.000	0.880	0.062	0.001
2	0.920	0.049	0.000	0.880	0.063	0.001
3	0.920	0.051	0.000	0.875	0.064	0.001
4	0.905	0.055	0.000	0.875	0.064	0.001
5	0.910	0.054	0.000	0.880	0.064	0.001
6	0.915	0.052	0.000	0.885	0.063	0.001
7	0.910	0.054	0.000	0.880	0.064	0.001
8	0.910	0.054	0.000	0.880	0.064	0.001
9	0.910	0.053	0.000	0.880	0.064	0.001
10	0.910	0.054	0.000	0.885	0.063	0.001
11	0.910	0.054	0.000	0.885	0.063	0.001
12	0.910	0.053	0.000	0.880	0.063	0.001
13	0.910	0.054	0.000	0.880	0.064	0.001
14	0.910	0.054	0.000	0.885	0.063	0.001
15	0.905	0.055	0.000	0.885	0.063	0.001
16	0.910	0.054	0.000	0.880	0.064	0.001
17	0.910	0.054	0.000	0.880	0.064	0.001
18	0.905	0.055	0.000	0.880	0.063	0.001
19	0.910	0.053	0.000	0.885	0.063	0.001
20	0.915	0.052	0.000	0.895	0.059	0.001

(c)	Atopic vs non-atopic asthma			Controlled vs uncontrolled asthma		
Steps (k)	Area under the curve	Standard error	Asymptomatic Significance	Area under the curve	Standard error	Asymptomatic Significance
1	0.865	0.066	0.001	0.850	0.069	0.002
2	0.865	0.067	0.001	0.850	0.069	0.002
3	0.860	0.069	0.002	0.840	0.072	0.003
4	0.880	0.063	0.001	0.850	0.069	0.002
5	0.875	0.064	0.001	0.855	0.068	0.002
6	0.880	0.063	0.001	0.855	0.068	0.002
7	0.880	0.063	0.001	0.850	0.069	0.002
8	0.875	0.064	0.001	0.855	0.068	0.002
9	0.875	0.064	0.001	0.850	0.069	0.002
10	0.880	0.063	0.001	0.855	0.068	0.002
11	0.875	0.064	0.001	0.850	0.069	0.002
12	0.880	0.063	0.001	0.855	0.068	0.002
13	0.870	0.065	0.001	0.850	0.069	0.002
14	0.885	0.061	0.001	0.855	0.068	0.002
15	0.890	0.060	0.001	0.855	0.068	0.002
16	0.885	0.062	0.001	0.855	0.068	0.002
17	0.880	0.063	0.001	0.850	0.069	0.002
18	0.875	0.064	0.001	0.850	0.069	0.002
19	0.880	0.063	0.001	0.850	0.069	0.002
20	0.880	0.063	0.001	0.845	0.070	0.002

Supplementary material 3. Partial autocorrelation graphs for (a) Heart rate variability in cirrhotic patients (b) Inter-breath-interval variability in patients with asthma (c) Body temperature fluctuations in cirrhotic patients.

(a)

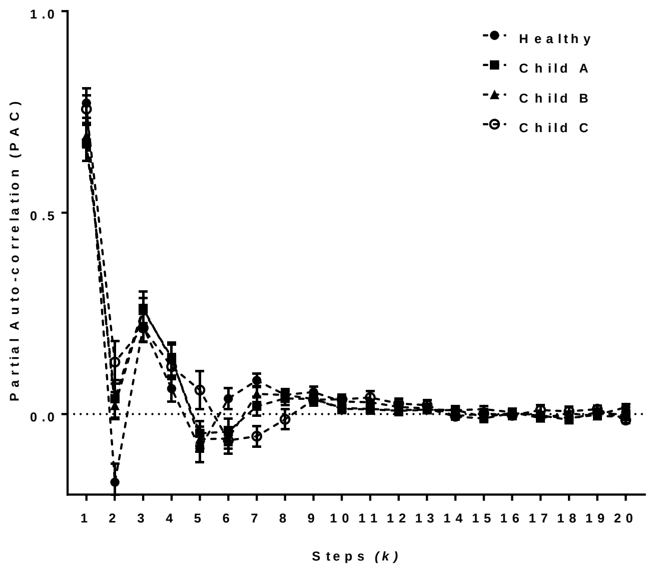

(b)

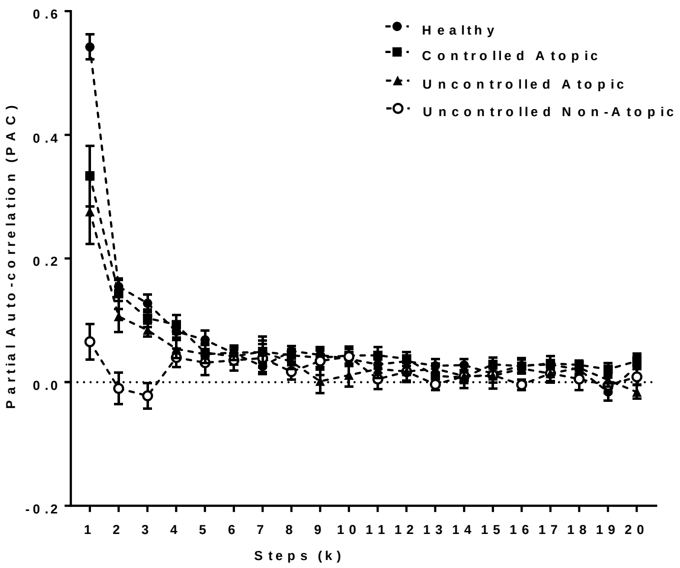

(c)

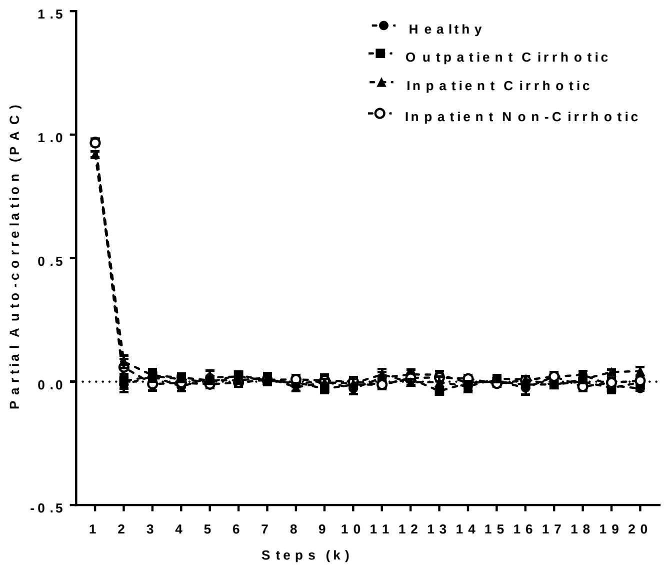

Supplement: Supplementary file 1 [file Data_Sheet_1.pdf]
